# Changes in Acidity, TSS, and Sugar Content at Different Storage Periods of the Postharvest Mango (*Mangifera indica* L.) Influenced by Bavistin DF

**DOI:** 10.1155/2013/939385

**Published:** 2013-12-29

**Authors:** Md. Khairul Islam, M. Z. H. Khan, M. A. R. Sarkar, Nurul Absar, S. K. Sarkar

**Affiliations:** ^1^Department of Crop Science and Technology, University of Rajshahi, Rajshahi 6205, Bangladesh; ^2^Department of Chemical Engineering, Jessore Science and Technology University, Jessore 7408, Bangladesh; ^3^Department of Genetic Engineering and Biotechnology, Jessore Science and Technology University, Jessore 7408, Bangladesh; ^4^Department of Biochemistry and Molecular Biology, University of Rajshahi, Rajshahi 6205, Bangladesh; ^5^Department of Biochemistry and Biotechnology, University of Science and Technology Chittagong, Chittagong 4202, Bangladesh

## Abstract

A detailed study was carried out with the postharvest mangoes (namely, the Langra and the Khirshapat) treated with different levels of Bavistin DF (BDF) solution (namely, 250, 500, and 750 ppm) for obtaining results on biochemical changes as well as storability of postharvest mango. The experiment was laid out in randomized complete block design with three replicates. The results of the experiments exhibited that only the single effect of varieties was found to be significant in most of the parameters studied. The Langra enriched a greater quantity of titratable acidity and total soluble solid (TSS) at 3rd day, over the Khirshapat. On the other hand, Khirshapat showed increased pulp pH and TSS at all the storage duration. The results explored that some physicochemical properties, namely, pulp pH, TSS, sugar (total, reducing, and nonreducing), and titratable acidity along with shelf life drastically decreased from untreated mangoes. Bavistin DF with the doses of 750 ppm showed better results in delaying the changes in physicochemical properties and extended shelf life.

## 1. Introduction

The mango is a commercial crop in many countries of the Southeast Asia, namely, India, Pakistan, the Philippines, Indonesia, Malaysia, Thailand, Burma, Sri Lanka, and Bangladesh [1]. Mango ranks third among the tropical fruits grown in the world with a total production of 23.87 million tons [2], to which Bangladesh contributed by only 0.64 million tons [3]. It is considered as a fruit crop for home consumption. It is also gaining rapid popularity in the Middle East, South East Africa, South Africa, Florida, Israel, and Australia. Among the fruits, mango ranks first in terms of area and third in terms of production in Bangladesh [4].

Nutritionally, it contains substantial quantity of appreciable *β* carotene, vitamin C, and dietary fibre [5] as well as soluble sugars and different minerals which are used as good sources of nutrition, and readily available and easily assumable in human body [6], and therefore it is capable to prevent many deficiency diseases [7, 8]. Approximately 30–50% fruits go wasted during postharvest handling, storage, and ripening [9]. Among the fruits mango manifested high postharvest losses because of its high perishability and climacteric pattern of respiration. The marketability of this perishable fruit is closely linked with the development of suitable technology which reduces the losses at different stages of harvesting and storage condition.

Losses in terms of quality and quantity of fruits occur at all stages in the postharvest system from harvesting to consumption. Reliable statistical data are inadequate especially in Bangladesh to indicate the magnitude of postharvest losses of mango. Singh et al. [6] reported that the postharvest losses of mango fruit in India due to microbial decay ranged from 20 to 33%. Quality mangoes are produced in northwestern part of Bangladesh, of which about 35–38% of postharvest losses are caused due to inefficient handling during its transportation, storage, and marketing.

Mango always decays after harvest, and postharvest losses can be considerably reduced by applying improved storage technology and prolonging the shelf life of fruits. Several researchers used Bavistin DF (BDF) for controlling spoilage of different fruits [10, 11]. The efficacy of Bavistin against the fruit rot pathogen was reported by several workers [12, 13]. Although Bavistin was observed to be the most effective treatment, there are numerous reports of the negative effects of using chemicals on farm income and the health of farm workers. Toxic contamination to the environment, particularly in developing countries, has also been reported.

These treatments strongly impede in ethylene synthesis that resulted in low respiration and delay ripening. These materials also reduced the losses and prolonged the shelf life of mango [14]. In addition, fungicidal treatments like Bavistin DF (BDF) are also excellent ethylene inhibitors. These treatments performed effectively in reduction of postharvest decay and extension of shelf life of mango [15]. Apparently, these treatments deteriorate the qualities of fruits to some extent, but the reduction of losses and extension of postharvest life of mango will help to increase the market price in the off seasons which play a good role in the economic development.

In this present investigation we tried to study the behavioral pattern of physicochemical properties of postharvest mango in the storage conditions. Especially the effects of BDF on pH, tritability, and sugar contents were studied in detail. It was also aimed to find out a desirable technology for extension of storage of mango.

## 2. Materials and Methods

### 2.1. Experimental Materials

The two mango varieties, namely, Langra and Khirshapath, were selected as experimental materials. The mango varieties undertaken for investigation were collected from mango grower of Kansart, Shibganj Upazila of Chapainowabgonj district and Charghat Upazila of Rajshahi district, and other material used as postharvest treatments, namely, Bavistin DF (BDF), were collected from Royal Scientific store at cooperative market, Rajshahi City market. The experiment consisted of two factors and was conducted in Randomized Complete Block Design (RCBD) with three replicates.

The experiment was laid out in a randomized complete block design with three replicates. The postharvest treated fruits were assigned at random in each replication. The required numbers of unblemished physically similar, more or less uniform size, shape and color fruits for the experiment were harvested manually from each plant of the varieties, Langra and Khirshapath. The fruits were carefully selected during harvest. The skin of fruits was cleared with the help of a cloth just after harvesting.

### 2.2. Preparation of Bavistin DF (BDF) Solution

The 250, 500, and 750 ppm of BDF solution were prepared by dissolving 250, 500, and 750 mg of BDF (commercial product) in one litre of distilled water. It is noted that the active ingredient of BDF is carbendazim whose initial concentration is 500 g/kg, that is, 50% (product name—Bavistin 50 DF). The fruits of both varieties were dipped into the BDF solution for a period of 5 minutes. Care was taken to ensure enough quantity of BDF being absorbed by the fruits and stored at ambient condition on brown paper.

### 2.3. Titratable Acidity in Mango Pulp

Titratable acidity of mango pulp was determined by the method [16].

#### 2.3.1. Reagents

The following reagents were used for determination of titrable acidity:

(i) standard NaOH solution (0.1 N),
(ii) 1% methyl red.

#### 2.3.2. Extraction of Mango Juice

Ten g of mango pulp was taken in a 100 mL beaker and then it was homogenized with distilled water in a blender. The blended materials were then filtered and transferred to a 100 mL volumetric flask, and the volume was made up to the mark with distilled water.

#### 2.3.3. Titration Procedure

Ten mL of pulp solution was taken in conical flask. Two to three drops of phenolphthalein indicator was added, and then the conical flask was shaken vigorously. It was then filtrated immediately with 0.1 N NaOH solutions from a burette till a permanent pink color appeared. The volume of NaOH solution required for titration was recorded. Percent titratable acidity was calculated by using the following formula:

(1) $$\begin{array}{c} \%\text{Titratable}\;\text{acidity}=\left(\frac{T\times N\times V1\times E}{V2\times W\times 1000}\right)\times 100, \end{array}$$

where *T* is the titre, *N* is the normality of NaOH, *V*1 is the volume made up, *E* is the equivalent weight of acid, *V*2 is the volume of extract, and *W* is the weight of sample.

### 2.4. Determination of Total Sugar Content of Mango Pulp

Total sugar content of mango pulp was determined calorimetrically by the anthrone method [17].

#### 2.4.1. Reagents

The following reagents were used for determination of total sugar:

- anthrone reagent: the reagent was prepared by dissolving 2 g of anthrone in one litre of concentrated H_2_SO_4_,
- standard glucose solution: a standard solution of glucose was prepared by dissolving 10 mg of glucose in 100 mL of distilled water.

#### 2.4.2. Extraction of Sugar from Mango Pulp

Four g of mango pulp was cut into small pieces and immediately plunged into boiling ethyl alcohol and was allowed to boil for 5 to 10 minutes (5 to 10 mL of alcohol was used per gram of pulp). The extract was cooled and crushed thoroughly in a mortar with pestle. Then the extract was filtered through two layers of muslin cloths and the ground tissue was reextracted for three minutes in hot 80% alcohol, using 2 to 3 mL of alcohol per gram of tissue. The second extraction ensured complete removal of alcohol soluble substances. The extract was cooled and passed through two layers of muslin cloth. Both of the extracts were filtered through Whatmann no. 41 filter paper.

The volume of the extract was evaporated to about 25% (1/4) of the volume over a steam bath and cooled. This reduced volume of the extract was transferred to a 100 mL volumetric flask and it was made up to the mark with distilled water.

#### 2.4.3. Procedure

Aliquot of 1 mL of pulp extract was pipetted into test tubes and 4 mL of the anthrone reagent was added to each of this solution and mixed well. Glass marbles were placed on top of each test tube to prevent loss of water through evaporation. Then the tubes were placed in a boiling water bath for 10 minutes and then cooled. A reagent blank was prepared by taking 1 mL of water and 4 mL of anthrone reagent in a tube and treated similarly. The absorbance of blue green solution was measured at 680 nm in a colorimeter.

A standard curve of glucose was prepared by taking 0.0, 0.1, 0.2, 0.4, 0.6, 0.8, and 1.0 mL of standard glucose solution in different test tubes containing 0.0, 10, 20, 40, 60, 80, and 100 *μ*g of glucose, respectively, and the volume was made up to 1 mL with distilled water. Then 4 mL of anthrone reagent was added to each test tube and mixed well. All these solutions were treated similarly as described above. The absorbance was measured at 680 nm using the blank containing 1 mL of water and 4 mL of another reagent.

The amount of total sugar present in the extract was calculated from the standard curve of glucose. Finally, the percentage of total sugar was determined by using the following formula:

(2) %Total  sugar  (g/100 g  of  mango)  =(Quantity  of  sugar  obtainedWeight  of  sample)×100

*t* of acid, *V*2 = volume of extract, *W* = weight of sample.

### 2.5. Determination of Reducing Sugar of Mango Pulp

Reducing sugar content of mango was determined by dinitrosalicylic acid method [18]. Reducing sugar content of mango was determined by dinitrosalicylic acid method. Reagent used: (i) Dinitrosalicylic acid (DNS) solution: Simultaneously, 1.0 g of DNS, 200 mg of crystalline phenol, and 50 mg of sodium sulphite were placed in a beaker and mixed with 100 mL of 1% NaOH by stirring. When it was needed to store, sodium sulphite was added just before use.

## 3. Results and Discussion

### 3.1. Titratable Acidity

Variation between varieties means in connection with titratable acidity was perceived to be highly significant at different days after storage (Table 1). At various days of storage, the Langra was noticed to be a higher producer of titratable acidity as compared to the Khirshapat. Titratable acidity abated with the advancement of storage period. The abating trend was hastil from the initial to 3rd day and, thereafter, it was slower. At initial day, higher (3.77%) was derived from the Langra while lower (2.47%) was noticed from the Khirshapat. At 12th day, higher (0.31%) was recorded from the Langra while, the Khirshapat gave lower amount (0.24%). The decreasing trend of titratable acidity during storage period was reported by Upadhyay et al. [19] also found the similar results. According to them, acidity was reduced during storage growth on attainment of maturity and ripening. The results of the present investigation might be possibly due to genetically dissimilarities between two varieties.

**Figure 1:**
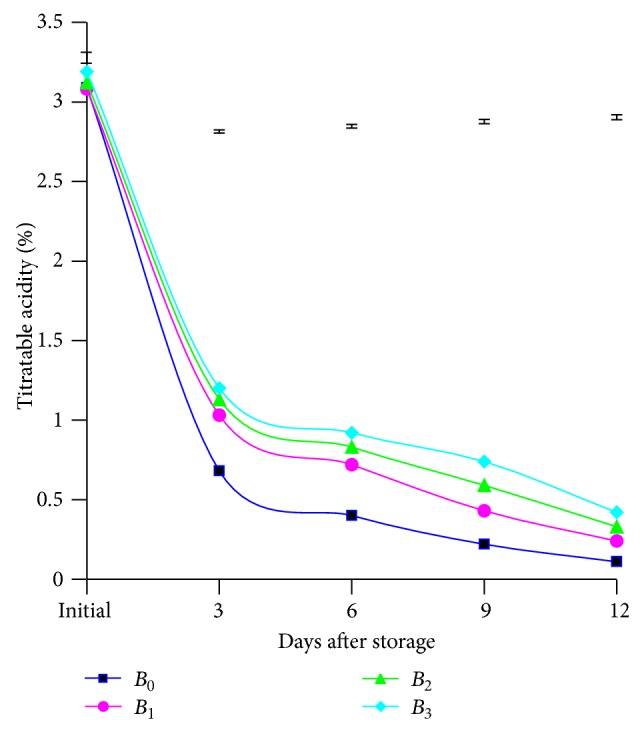
Status of titratable acidity of mango pulp as influenced by different doses of BDF at different days after storage. Vertical bars represent LSD at 0.05 level.

Different doses of BDF solution imposed to this investigation in terms of titratable acidity showed significant variation among the means at various days of storage. At different days of storage, titratable acid content diminished hastily from initial to 3 days, and then it diminished steadily (Figure 1). In all the storage period, higher titratable acidity (3.19, 1.20, 0.92, 0.74, and 0.42%) was derived from *B*_3_ treatment from initial to 12th days followed by 3.09, 0.68, 0.40, 0.22, and 0.11% from untreated mangoes. These results are supported by the findings of Ahmed and Singh [20]. These phenomena happening might be possible due to *B*_3_ treatment delayed ripening that caused lower diminishing trend of titratable acidity while control treatment caused ripening fast resulting in high decreasing trend of titrable acid content.

The combined effect of varieties and different doses of BDF solution in relation to titratable acidity of mango pulp demonstrated significant variation at different days after storage except initial days. At different days of storage, there appeared a decreasing trend of titratable acid content with the rising of storage period. At the 6th day, the highest (1.00%) quantity was recorded from the treatment combination of *V*_1_ *B*_3_ and the lowest acid concentration (0.30%) was recorded from the treatment combination of *V*_2_ *B*_0_ (table is not mentioned here). This occurrence might be probably due to the reduction of acid oxidation at *V*_2_ *B*_3_ combination as well as genetic variation in between varieties.

### 3.2. Pulp pH

**Figure 2:**
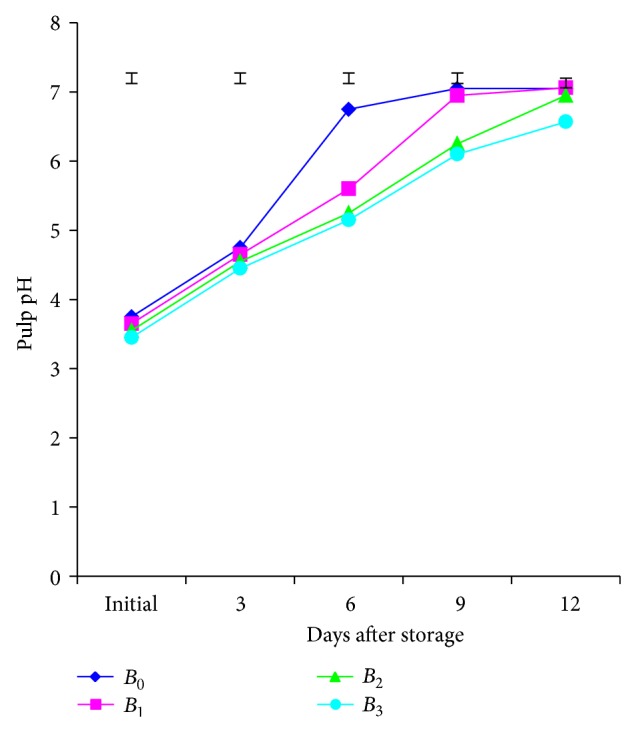
Pulp pH of mango pulp as influenced by different doses of BDF at different days after storage. Vertical bars represent LSD at 0.05.

The analysis of variance between the varieties exhibited significant variation in terms of pulp pH of mango at different days after storage except at 6th day (Figure 2). At various days of storage, a growing up trend of pulp pH with the increase of storage period was observed. In each storage period, pulp pH was found more in the Khirshapat compared to the Langra. Higher pulp pH (6.96) was noted from the Khirshapat at 12th day whereas lower (6.85) was noted from the Langra. The growing up trend of pulp pH was also observed by Shahjahan et al. [21]. This phenomenon might be possible due to oxidation of acid during storage resulting in higher pH and also might have been genetical dissimilarities between varieties.

Different doses of BDF solution subjected to this trial showed significant variation in pulp pH at different days after storage. The results indicated that the growing up trend of pulp pH was perceived from different treated and untreated mangoes at various days of storage (Figure 2). Pulp pH was higher in control at all stages of storage followed by the fruits treated with *B*_1_, *B*_2_, and *B*_3_ treatments, respectively. The pH value of mango pulp was higher (7.05) in control which was statistically at par with *B*_1_ and *B*_2_ treatment whereas the fruits treated with *B*_3_ produced lower (6.57) value at 12th day. The results of the present investigation at *B*_3_ treatment interrupted the loss of acid oxidation resulting in lower pH value. These results are in agreement with the findings of Ahmed and Singh [20].

The combined effect of varieties and different doses of BDF solution imposed to this study in pulp pH were noticed to be nonsignificant at different days after storage. There appeared a slightly rising trend of pulp pH from various treatment combinations at different days of storage. At 6th day, the highest (6.90) pH value was reported from the treatment combination of *V*_2_ *B*_0_ which was statistically at par with *V*_1_ *B*_0_ and the lowest (6.70) was reported from the treatment of combination of *V*_1_ *B*_3_, respectively.

### 3.3. Total Soluble Solid (Brix %) Content

Statistically highly significant variation was observed in TSS content between two varieties at different days after storage. The results exhibited that TSS content of mango pulp developed in a continuous stream with the expansion of storage period. The developing trend was hastily from initial to 6th day, thereafter; it increased slower. From initial to 6th day, the Khirshapat enriched a better amount of TSS than the Langra, but, after 6th day, Langra performed better than the Khirshapat up to 12th day. At 9th day, higher (17.90%) TSS quantity was noted from the Khirshapat and lower (17.00%) was noted from the Langra (Table 2). Higher percentage of TSS during storage in the mango was reported by Singh [20]. Absar et al. [22] reported that TSS was increased with maturity of mango fruit. But, they found highest TSS in the Langra. These might be possible due to genetic differences between varieties.

**Figure 3:**
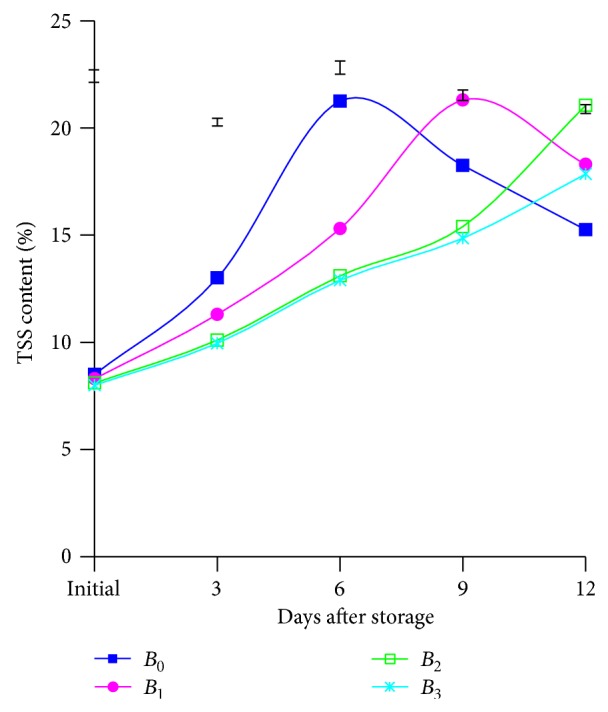
Effect of different doses of BDF on total sugar content of mango pulp at different days after storage. Vertical bars represent LSD at 0.05 level.

Different doses of BDF solution implied to the postharvest mangoes in this study were noticed to be significant in terms of TSS content at different days after storage. At different days of storage, the results showed that TSS accumulation increased with the increase of storage duration. It also explored that TSS content was hastily grown up from untreated mangoes from initial to 6th day and then it came down significantly (Figure 3). The other treatment namely, *B*_1_, also increasingly produced TSS from initial to 9th day, and thereafter it decreased sharply. Mango fruits treated with *B*_2_ also produced more or less similar enhancing trend from initial to 12th days. But the fruits treated with *B*_3_ treatment gave very slower motion in TSS accumulation at various days after storage. The highest (21.25 and 21.30%) accumulation of TSS content was perceived from *B*_0_ and *B*_1_ treatment at 6 and 9th days while the lowest (12.90 and 14.85%) was noted from *B*_3_ treatment (Figure 3). The results of the present studies are strongly supported by the findings of Dhemre and Waskar [23] also found the similar results.

These happened possibly due to ripening condition resulting in maximizing TSS accumulation in control and 750 ppm of BDF solution resisted in ethylene synthesis that caused delay ripening and ultimately in lower TSS accumulation. It also revealed that TSS accumulation is strongly related to ripening and it caused falling off owing to decaying.

The combined effect of varieties and applied different doses of BDF solution in connection with TSS content were perceived to be significant at different days after storage except initial and 3rd day. An enhancing behavior of TSS content at different days after storage was exposited. The highest accumulation (22.00, 21.80, and 21.60%) was obtained from the treatment combination of *V*_1_ *B*_0_, *V*_1_ *B*_1_, and *V*_1_ *B*_2_ at 6, 9 and 12th days, while the lowest value (13.20, 14.50, and 17.50%) was notified from the treatment combination of *V*_2_ *B*_3_ (table is not presented here), respectively.

### 3.4. Total Sugar Content

Highly significant variation was manifested between both varieties means in terms of total sugar content of mango pulp at different days after storage. It elucidated that TSC gathered in a continuous stream with expanding of storage duration. This gathering trend was more or less hastil from initial to 9th days in both the varieties; thereafter; it expanded slightly slower. At all days of storage, the Khirshapat produced more quantity of TSC than the Langra. At initial day, the Khirshapat had higher (6.09%) while; the Langra provided lower (5.57%). At 12th day, it gave higher quantity (19.64%) and lower (19.07%) was noted in the Langra. Upadhyay et al. [19] reported that total sugar content was expanded gradually, when stored for 6 days at room temperature. Sugar content increased during ripening. These results are in conformity with the findings of Shahjahan et al. [21]. The increase in TSC might be possible due to conversion of complex starch or carbohydrate into simple compound.

**Figure 4:**
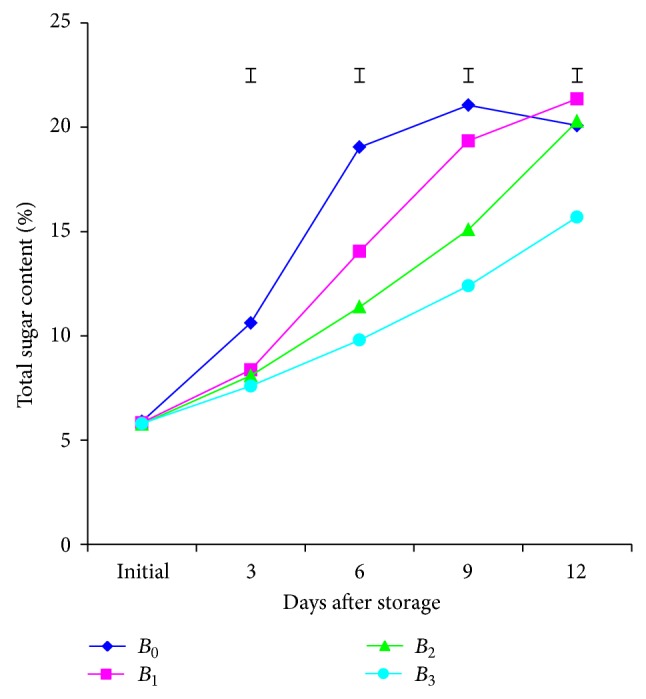
Effect of different doses of BDF on TSS of mango pulp at different days after storage. Vertical bars represent LSD at 0.05 level.

Different doses of BDF solution subjected to the investigation in connection with total sugar content of mango pulp demonstrated significant variation at different days after storage except initial day. At different days, the results found that TSC increased hastily with expanding of storage period (Figure 4). The increasing trend was very swift in untreated mango followed by other treatments, namely *B*_1_, *B*_2_ and *B*_3_ treatment, respectively. The highest quantity of TSC (21.06 and 21.36%) was obtained from control and *B*_1_ treated mangoes at 9 and 12th days, while the lowest (12.40% and 15.70%) was reported from *B*_3_ treatment. The results of the present investigation are in conformity with the reports of Dhemre and Waskar [23]. The enhancing trend of total sugar at untreated mangoes might be perhaps due to breaking down of complex carbohydrate into simple compound but *B*_3_ treatment made delay ripening at storage period.

The combined effect of varieties and implied different doses of BDF solution in this study in terms of total sugar content of mango pulp exhibited nonsignificant variation at different days after storage. The results indicated that total sugar content progressively accumulated with the advance of storage period (table is not mentioned here). At 9th day, the maximum (21.31%) quantity of TSC was formed from the treatment combination of *V*_2_ *B*_0_ while the minimum (12.10%) was formed from the treatment combination of *V*_1_ *B*_3_, respectively.

### 3.5. Reducing Sugar Content

Analysis of variance showed significant effect on reducing sugar content of mango pulp at different days of storage except at 6th day. The results showed an increasing trend of reducing sugar with the progress of storage period. It also annotated that the Khirshapat was found better in enriching of reducing sugar than the Langra at different days of storage. Higher (5.47%) quantity of this sugar was observed from the Khirshapat while lower (5.20%) quantity was noticed from the Langra at 12th day of storage (Table 3). These results are in agreement with the findings of Upadhyay et al. [19]. Casttrillo and Bermudez [24] stated that reducing sugar content was augmented during storage period. Khirshapat providing more reducing sugar might be possibly due to genetical variation in both varieties.

Different doses of BDF subjected to this study were perceived to be significant in respect of reducing sugar content of mango pulp at different storage periods except initial day. The results explained that reducing sugar of mango pulp was increased progressively at different days of storage. It also stated that untreated mangoes were better in forming of reducing sugar as compared to other treatments. Control was found to be a more effective producer of reducing sugar up to 9th day and then it came down owing to starting decay. At 12th day, the maximum (6.27%) amount of reducing sugar was obtained from *B*_1_ treatment and the lowest (4.32%) was obtained at *B*_3_ treatment (Table 3). The results of the present study are in inconformity with the findings of Dhemre and Waskar [23]. Lower increasing trend of reducing sugar content treated with *B*_3_ treatment might be possibly due to delayed ripening that resulted in lesser conversion of carbohydrates into simple's molecules.

The combined effect of varieties and different doses of BDF solution of mango pulp demonstrated nonsignificant variation in terms of reducing sugar content of mango pulp at different days after storage. The results exposited that reducing sugar content grew up progressively at three-day interval up to 9th day; thereafter, it came down from the treatment combination of *V*_2_ *B*_0_. At 9th day, the highest (6.44%) quantity was noticed from the treatment combination of *V*_2_ *B*_0_ and the lowest (3.90%) was noticed from *V*_1_ *B*_3_ (Table 4).

### 3.6. Nonreducing Sugar Content

The variation between the varieties means demonstrated highly significant in respect of nonreducing sugar content at different days after storage. The results were noticed to be an enhanchable trend of nonreducing sugar content at different days of storage. At all the days, it revealed that the Khirshapat was observed to be much better than the Langra in receiving of nonreducing sugar content (Table 3). At 12th day, higher (14.20%) amount of nonreducing sugar was recorded from the Khirshapat and lower (13.88%) amount was recorded from the Langra.

Different doses of BDF solution imposed to this trial were noticed to be a significant variation in connection with nonreducing sugar content of mango pulp at different days after storage except at initial stage. The results stated that nonreducing sugar content of mango pulp was formed progressively at various days. It denoted that untreated fruits were noticed to be better in achieving more quantity of nonreducing sugar followed by the other treatments. This increasing trend was markedly up to 9th day, and thereafter it increased slowly owing to becoming hackneyed. Lower rising trend was perceived from the fruit treated with *B*_3_ treatment. At 12th day, the highest result (15.28%) was recorded from control and lowest value (11.39%) was recorded from *B*_3_ treatment (Table 3). These events might be probably due to *B*_3_ treatment retarded ethylene synthesis of mango pulp resulting in delayed ripening and little amount of nonreducing sugar achieving. These results are in agreement with the statement of Ahmed and Singh [20].

The combined effect of varieties and different doses of BDF solution exhibited nonsignificant in terms of nonreducing sugar content of mango pulp at different days after storage. The results showed a mild growing up trend of nonreducing sugar from different treatment combinations at various days (Table 4). At 9th day, the highest (14.87%) quantity of nonreducing sugar was notified from the treatment combination of *V*_2_ *B*_0_ while the lowest (8.42%) was notified from the treatment combination of *V*_1_ *B*_3_, respectively.

## 4. Conclusion

Different doses of BDF solution imposed to this investigation in terms of titratable acidity showed significant variation among the means at various days of storage. The combined effect of varieties and different doses of BDF solution imposed to this study in pulp pH were noticed to be nonsignificant at different days after storage. A slightly rising trend of pulp pH from various treatment combinations at different days of storage appeared. At different days of storage, the results showed that TSS accumulation increased with the increase of storage duration. It also explored that TSS content was hastily grown up from untreated mangoes from initial to 6th day, and then it came down significantly. The combined effect of varieties and different doses of BDF solution exhibited nonsignificant in terms of nonreducing sugar content of mango pulp at different days after storage. The results showed a mild growing up trend of nonreducing sugar from different treatment combinations at various days.

## Figures and Tables

**Table 1:** Changes of titratable acidity and pH of postharvest mango pulp in varieties during storage at room temperature.

Treatments	Titratable acidity (%) at different days					Pulp pH at different days				
Variety (*V*)	Initial	3	6	9	12	Initial	3	6	9	12

*V*_1_	3.77^a^	1.10^a^	0.78^a^	0.55^a^	0.31^a^	3.55^b^	4.55^b^	5.65	6.53^b^	6.85^b^
*V*_2_	2.47^b^	0.92^b^	0.65^b^	0.43^b^	0.24^b^	3.65^a^	4.65^a^	5.73	6.65^a^	6.96^a^

Level of significance	∗∗∗	∗∗∗	∗∗∗	∗∗∗	∗∗∗	∗	∗	NS	∗	∗

Note: *V*_1_ = Langra; *V*_2_ = Khirshapat; ∗ indicates at 5% level; ∗∗ indicate at 1% level; ∗∗∗ indicate at 0.1% level.

**Table 2:** Pattern of total soluble solid and total sugar content of postharvest mango pulp between varieties during storage at ambient condition.

Treatments	TSS content (%) at different days					Total sugar content (%) at different days				
Variety (*V*)	Initial	3	6	9	12	Initial	3	6	9	12

*V*_1_	7.73^b^	10.60^b^	15.75^a^	17.90^a^	18.65^a^	5.57^b^	8.39^b^	13.29^b^	16.70^b^	19.07^b^
*V*_2_	8.73^a^	11.58^a^	15.53^b^	17.00^b^	17.58^b^	6.09^a^	8.95^a^	13.85^a^	17.26^a^	19.64^a^

Level of significance	∗∗∗	∗∗∗	∗∗	∗∗∗	∗∗∗	∗∗∗	∗∗∗	∗∗∗	∗∗∗	∗∗∗

**Table 3:** Behavior of reducing and nonreducing sugar content of postharvest mango pulp in varieties, influenced by different doses of Bavistin DF solution during storage condition.

Treatments	Reducing sugar content (%) at different days					Nonreducing sugar content (%) at different days				
Variety (*V*)	Initial	3	6	9	12	Initial	3	6	9	12

*V*_1_	1.45^b^	2.17^b^	3.82^b^	5.17^b^	5.20^b^	4.13^b^	6.22^b^	9.47^b^	11.53^b^	13.88^b^
*V*_2_	1.72^a^	2.46^a^	4.11^a^	5.44^a^	5.47^a^	4.32^a^	6.49^a^	9.74^a^	11.82^a^	14.20^a^

Level of significance	∗∗	∗∗∗	∗∗∗	∗∗∗	∗∗∗	∗∗∗	∗∗∗	∗∗∗	∗∗∗	∗∗∗

Treatments (*B*)										
*B*_0_	1.63	3.10^a^	6.10^a^	6.30^a^	4.80^c^	4.28	7.53^a^	12.95^a^	14.77^a^	15.28^a^
*B*_1_	1.62	2.20^b^	3.90^b^	6.15^b^	6.27^a^	4.24	6.17^b^	10.15^b^	13.20^b^	15.09^b^
*B*_2_	1.56	2.05^c^	3.25^c^	4.95^c^	5.95^b^	4.21	6.04^c^	8.14^c^	10.14^c^	14.39^c^
*B*_3_	1.53	1.92^d^	2.62^d^	3.82^d^	4.32^d^	4.18	5.69^d^	7.19^d^	8.59^d^	11.39^d^

Level of significance	NS	∗∗∗	∗∗∗	∗∗∗	∗∗∗	NS	∗∗∗	∗∗∗	∗∗∗	∗∗∗

In a column values having the same letter(s) do not differ significantly as per DMRT at 5% level.

**Table 4:** Combined effects of varieties and different doses of Bavistin DF solution on reducing and nonreducing sugar content of postharvest mango pulp at ambient condition.

Treatments combination	Reducing sugar content (%) at different days					Nonreducing sugar content (%) at different days				
Varieties × treatments	Initial	3	6	9	12	Initial	3	6	9	12

*V*_1_ *B*_0_	1.48	2.95	5.95	6.15	4.65	4.17	7.42	12.85	14.67	15.18
*V*_1_ *B*_1_	1.48	2.05	3.75	6.05	6.17	4.13	6.06	10.04	13.04	14.92
*V*_1_ *B*_2_	1.42	1.90	3.10	4.80	5.80	4.11	5.88	7.98	9.98	14.18
*V*_1_ *B*_3_	1.40	1.78	2.48	3.68	4.18	4.10	5.52	7.02	8.42	11.22

*V*_2_ *B*_0_	1.77	3.24	6.24	6.44	4.94	4.38	7.63	13.05	14.87	15.38
*V*_2_ *B*_1_	1.75	2.35	4.05	6.25	6.37	4.35	6.28	10.26	13.36	15.26
*V*_2_ *B*_2_	1.70	2.20	3.40	5.10	6.10	4.30	6.20	8.30	10.30	14.60
*V*_2_ *B*_3_	1.65	2.05	2.75	3.95	4.45	4.25	5.85	7.35	8.75	11.55

Level of significance	NS	NS	NS	NS	NS	NS	NS	NS	NS	NS

CV%	6.71	4.58	2.68	2.00	1.99	2.51	1.67	1.10	0.91	0.76

*B*_0_ = control; *B*_1_ = 250 ppm of BDF solution; *B*_2_ = 500 ppm of BDF solution; *B*_3_ = 750 ppm of BDF solution.
